# *Glucosamine-6-phosphate N-acetyltransferase* gene silencing by parental RNA interference in rice leaf folder, *Cnaphalocrocis medinalis* (Lepidoptera: Pyralidae)

**DOI:** 10.1038/s41598-022-06193-9

**Published:** 2022-02-08

**Authors:** Muhammad Shakeel, Juan Du, Shang-Wei Li, Yuan-Jin Zhou, Naeem Sarwar, Xiaolan Guo

**Affiliations:** 1 Provincial Key Laboratory for Agricultural Pest Management of Mountainous Regions, Institute of Entomology, Guizhou University, Guiyang, 550025 Guizhou China; 2 Department of Agronomy, Bahauddin Zakariya University, Multan, 60800 Pakistan; 3 College of Forestry, Guizhou University, Huaxi, 550025 China

**Keywords:** Molecular biology, Plant sciences, Zoology

## Abstract

Parental RNAi (pRNAi) is a response of RNA interference in which treated insect pests progenies showed a gene silencing phenotypes. pRNAi of *CmGNA* gene has been studied in *Cnaphalocrocis medinalis* via injection. Our results showed significant reduction in ovulation per female that was 26% and 35.26% in G1 and G2 generations, respectively. Significant reduction of hatched eggs per female were observed 23.53% and 45.26% as compared to control in G1–G2 generations, respectively. We also observed the significant variation in the sex ratio between female (40% and 53%) in G1–G2 generations, and in male (65%) in G1 generation as compared to control. Our results also demonstrated the significant larval mortality (63% and 55%) and pupal mortality (55% and 41%), and significant reduction of mRNA expression level in G1 and G2 generations. Our findings have confirmed that effectiveness of pRNAi induced silencing on the *CmGNA* target gene in G1–G2 generations of *C. medinalis*. These results suggested the potential role of pRNAi in insect pest resistance management strategies.

## Introduction

Rice (*Oryza sativa* L., family *Poaceae*) is the world's largest cereal crop that is widely cultivated around the globe. Rice has been accepted as a staple food for almost one-half of the human population in the world^1,2^. In Asia, almost 90% of people feed rice to fulfill their dietary requirements^3^. Rice considers as a staple food for more than 65% of Chinese population and is a subsistence crop provides income and support for rural communities^4^. Unfortunately, serious insect’s pest attack reduces rice production and its quality^5^. Over 100 insect pest species of rice have been recorded worldwide^6^. Out of these, almost 20 species are considered most injurious for rice crop that include leafhoppers, mole cricket, rice bugs, rice gall midges, rice mealy bug, stem borers, and rice leaf folder^7^.

Rice leaf folder (*Cnaphalocrocis medinalis)* is a major destructive rice insect pest that can cause severe grain yield losses worldwide. It is widely distributed in many rice growing countries of Asia, Africa, Australia and Oceania^8^. *C. medinalis* consists of complete metamorphosis that passes four different developmental stages such as egg, larva, pupa, and adult^9^. *C. medinalis* larvae have five stadiums that can damage at all the rice stages^10^. However, 4th larval stadium is considered most destructive for rice leaves^10^. Major characteristic of this insect pest is to scroll the leaves blades and scratch chlorophyll pigment inside them^11^. Scratched leaves become whitish, membranous, and wither that inhibits photosynthesis, and ultimately reduces crop productivity^12^. *C. medinalis* larvae can reduce 30 to 80% yield during epidemic situation^13^. *C. medinalis* is a migratory insect pest that possess 1–11 generations per year^14^. Existence of alternative plant hosts near paddy field create suitable environment to complete its multiple generations per year^15^. Shady places of paddy field and high humidity with high temperature are suitable for it growth and development^15^. At present, *C. medinalis* population often managed with extensive use of chemical insecticides^16^. However, misuse of insecticides drove insecticidal resistance, insect pest resurgence, dangerous to farmer’s health, toxic to environment, polluting underground water, and poisoning of Chinese food stuff^8^. Insecticidal resistance of *C. medinalis* was reported in Japan^17^, China^18^, and in India^19^. *C. medinalis* has developed high resistance against chlorantraniliprole, indoxacarb, monosultap, metaflumizone, chlorpyrifos, tebufenozide, and tebufenozide^20^. Furthermore, previous studies has reported that insecticidal resistance is due to physiological and behavioral modification that can minimize target sensitivity and increase detoxification in *C. medinalis*^21^.Therefore, it is crucial to identify safe and environmental friendly method to control this notorious insect pest. Chitin biosynthesis is present in insect and not found in vertebrates^22^. Therefore, we have considered to target chitin biosynthetic genes for control of *C. medinalis*.

Chitin (β-1,4-linked *N*-acetyl-d-glucosamines) is the 2nd most important biopolymer in nature after cellulose. It is produced by fungi, nematode, mollusks, protozoan, and arthropods^23^. Chitin is a major component of trachea, foregut, hindgut, peritrophic membrane, extracellular linings and embryonic cuticle of insects^24^. In insects, chitin has critical role in cuticle formation of an exoskeleton that plays a pivotal role in insect growth and development^25^. Many chitin synthesis associated genes have been studied in eggshells, ovaries, and exoskeleton of insects^26^. Therefore, silencing of chitin biosynthesis genes may result abnormal growth, body deformities, inhibit molting or even cause mortality^27^.

RNA interference (RNAi) has been recognized as an effective gene silencing tool in eukaryotic organisms^28^ that have conserved intracellular mode of action used to silence the gene expression^29^. Firstly, RNAi was described in *Caenorhabditis elegans*^30^. Later on, it was found in fungi, plants, animals, and in insects^31–34^. In insects, RNAi has been used to silence of gene expression against different insect pests such as *Tribolium castaneum*^35^, *Nilaparvata lugens*^36^, *Anopheles gambiae*^37^, *Diabrotica virgifera virgifera*^38^, *Spodoptera exigua*^39^, *Gryllus bimaculatus*^40^, *Manduca sexta*^41^, *Plutella xylostella*^42^, and Henosepilachna vigintioctopunctata sp.^43^. Gene silencing has been observed in late-instar larvae and adults of the lepidopteran *S. litura* via dsRNA injection^39,44^. Therefore, RNAi through dsRNA microinjection could be useful to silence the chitin biosynthesis genes in *C. medinalis*.

RNAi technology has been divided into three categories such as, larval/ nymphal/pupal RNAi (This technique has been used to study the gene expression in postembryonic stage and analyze the adult morphology on molecular basis in various organisms such as *T. castaneum*^45,46^, *Bombyx mori*^47^, *Schistocerca americana*^48^, *Blattella germanica*^49^, and *G*. *bimaculatus*^50–52^), embryonic RNAi (When dsRNA is incorporated into developing eggs in order to silence the target genes, the RNAi effects can observe in embryos such as *T. castaneum*^53^), and parental RNAi (Application of dsRNA into the body cavity via injection or ingestion that leads to gene silencing in offspring embryos^54,55^). Parental RNAi (pRNAi) effects were observed after silencing of zygotic genes in *T. castaneum* offspring^56^, gap genes, and *Krüppel* and *hunchback* genes in *Oncopeltus fasciatus*^57,58^. pRNAi effects were also recorded in cricket, *Gryllus bimaculatus*^59–61^. In wasp, *Nasonia vitripennis*, pRNAi was also found in injected pupae with dsRNA^62^. Phenotypic deformities such as canonical limb truncation and fusion of leg segments were observed in *Tetranychus urticae* after pRNAi^63^. In western corn rootworm, *D*. *virgifera virgifera*, less hatched eggs and incomplete larval development were observed in response by targeting embryonic developmental genes^64^. In addition, several chromatin remodeling ATPase genes such as iswi-1, iswi-2, mi-2, brahma, and hunchback genes were silenced using pRNAi in *Euschistus heros and D. virgifera virgifera*^65^. In grain aphid, *Sitobion avenae*, pRNAi effects were observed in many generations^66^. In *C. medinalis*, pRNAi effects were also found in three consecutive generation after silencing *CmHK* gene through dsRNA injection^67^. However, pRNAi effects of *CmGNA* gene has not studied in *C. medinalis.*

Glucosamine-6-phosphate N-acetyltransferase (GNA) is an essential enzyme of chitin biosynthesis pathway. Previously, *GNA* has been characterized in several eukaryotes such as, human^68^, rat^69,70^, pig^71^, *Saccharomyces cerevisiae*^72,73^, *Candida albicans*^74^, and *Aedes aegypti*^75^. It has been reported that *GNA* gene deletion in *S. cerevisiae* was lethal^73^. Therefore, we consider that silencing of *CmGNA* gene could be useful in pRNAi for the control of *C. medinalis*.

In RNAi assays, a dsGFP (green fluorescent protein derived dsRNA) has been used as an exogenous control for several insects, including *Spodoptera exigua*^76,77^, *Acyrthosiphon pisum*^78^, *Aedes aegypti*^79^, *Antheraea* sp.^80^, *Locusta migratoria*^81^, *Schistocerca gregaria*^82^, *Bactericerca cockerelli*^83^, and *Apis mellifera*^84–90^.

In this study, we carried out series of experiments to examine the effectiveness of pRNAi. We synthesized dsRNA and injected into different groups of larvae of *C. medinalis*. We observed that pRNAi of *CmGNA* had significant effects on eggs laying, hatched eggs, phenotypic deformities, moralities of larvae and pupae, male and female emergence rate, and reduced mRNA expression level of *CmGNA* gene in G1-G3 generation.

## Material and methods

### Rearing of *C. medinalis*

*Cnaphalocrocis medinalis* larvae were collected from rice growing field of Guiyang, Guizhou, China and reared at Entomological Institute of Guizhou University. The collected larvae were raised on fresh seedling of ShuHui-527 Chinese rice cultivar. The rearing chamber environment was kept at 75 ± 5% RH, 26 ± 1 °C, and 10:14 h dark: light photoperiod. Newly emerged adults were collected, paired (one male and female), and allowed them to mate for 3 to 4 days (Fig. S1). For this purpose, each adult’s pair was placed in oviposition box (5.1 cm long _ 3.8 cm wide _ and 2.9 cm high) with vented lids (Fig. S2). Each pair was fed with ddH_2_O diluted honey solution soaked in cotton plugs (Fig. S2). After oviposition, paired adults were removed and allowed eggs to hatch. The hatched larvae were collected and used for pRNAi experiments.

### Gene identification

*C. medinalis* transcriptome sequence was already described^91^. The *CmGNA* GenBank accession number was MN604261.

### RNA isolation, cDNA synthesis, and RT-PCR

RNA Isolation and cDNA Synthesis were performed as already described^67^. However, specific primers were designed (see list of primers in supplemental Table S1) for reverse transcription-polymerase chain reaction (RT-PCR) based on transcriptome of *C. medinalis*^91^. RT-PCR was carried out to confirm the expected size sequence of *CmGNA* gene. The RT-PCR system consisted of 20 μL reaction mixture containing 1 μL of each primer, 10 μL of 2 × Master Mix (Tsingke, Bejing, China), 1 μL of cDNA template, and 7 μL of ddH2O. The reactions conditions were as follows: initial denaturation at 94 °C for 30 s; 30 cycles of 94 °C for 30 s, 55 °C for 30 s, and 72 °C for 5 min, and a final extension of 72 °C for 10 min. The expected size was confirmed using agarose gel electrophoresis. The final products was then stored for further experiments.

### Double standard RNA (dsRNA) preparation

The dsRNA was synthesized in accordance with protocols as previously performed^67^. However, ds*CmGNA* and ds*GFP* sequence amplification and its synthesis were performed using different primers (see list of primers in Supplemental Table S1). The ds*GFP* was used as control.

The final purified product was quantified using NanoDrop 2000 spectrophotometer (Thermo Fisher, MA, USA).

### Parental RNAi (pRNAi) bioassay

Healthy 4th instar larvae of *C. medinalis* were selected for pRNAi. Seven groups were made along with control treatment and 20 larvae pooled per replication in each group. For dsRNA injection, 8th abdominal larval segment was selected at dorsal side along with the blood flow direction. The 0.5 μL (2 μg/μL) ds*CmGNA* and ds*GFP* were prepared and injected into the selected larvae. The treated larvae raised on ShuHui-527 fresh rice seedling under laboratory conditions as described above. To analyze the effects of pRNAi, newly emerged ten adults (five male and five female) from treated larvae were selected, paired and allowed them for oviposition. After oviposition, paired adults were removed from oviposition boxes and stored them in liquid nitrogen and kept at − 80 °C. Laid eggs were counted from each of mated pairs from G1 generation. A healthy female lays about nearly 135 eggs^92^. In order to estimate percentage laid eggs per female in control treatments, we compared control treatment laid eggs with 135 eggs and converted them into percentage. After hatched eggs, forty larvae were selected from each group (treated and control) and checked the mortality of both larvae and pupae. Forty pupae were selected from ds*CmGNA* and d*sGFP* treated each group and checked the percentage male and female emergence rate in G1, G2 and G3 generations, respectively. The mRNA transcriptional level was also measured using newly emerged adults from treatments. After measuring mRNA transcriptional level, newly emerged adults from G1 generation were used for G2, and G2 were used for G3 generation. All experiments were performed from at G1–G3 generations to verify the pRNAi effects.

### Quantitative real-time PCR (RT-qPCR)

RT-qPCR was used to measure the mRNA transcriptional level of *CmGNA* in G1-G3 generation adults. For this purpose, RNA isolation and cDNA synthesis from G1- G3 generation adults were carried out using protocols as mentioned above. The cDNA was used as templates to perform RT-qPCR. RT-qPCR reaction was performed using C^1000^ Thermal Cycler (Bio-Rad, CA, USA). The reaction system contained 20 μL reaction mixture included 1 μL cDNA, 1 μL of each primers, 10 μL 2 × iTaq Universal SYBR Green Supermix, and 7 μL ddH_2_O. RT-qPCR reaction was carried out under the following conditions: 95 °C for 2 min, followed by 40 cycles at 95 °C for 20 s, 56 °C for 20 s, and 72 °C for 30 s. *C. medinalis* actin gene (GenBank number. JN029806) was taken as the internal control. RT-qPCR primers were used as listed in Supplementary Table S1. The relative expression levels of *CmGNA* were calculated using the 2^−ΔΔCt^ method.

### Statistical analysis

Statistical Analysis were carried out with ANOVA (one-way analysis of variance) followed by LSD test using SPSS 22.0 (SPSS Inc. Chicago, IL, USA).

## Results

### RT-PCR and dsRNA synthesis

The cDNA sequence of *CmGNA* (GenBank Accession No. MN604261) consists 859 bp (Fig. S3). The ds*CmGNA* and ds*CmGFP* were clear and bright with 372 and 370 bps, respectively (Fig. S4). The ds*CmGNA* and ds*GFP* were quantified 3.1 μg/μL and 2.91 μg/μL, respectively.

### Effect of pRNAi on oviposition

**Figure 1:**
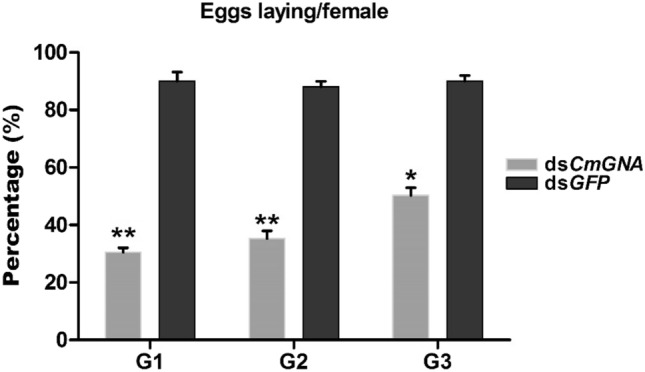
The percentage laid eggs per female in G1–G3 generations. Each point indicates the mean ± standard error in G1–G3 generations along with their control groups. Significant differences indicated by *(P < 0.05), **(P < 0.01).

In order to examine the pRNAi effects on oviposition, treated larvae were reared until they become adults. Paired adults were laid eggs in oviposition boxes. Harvested eggs from both treatments were counted. Counted eggs were then compared with control treatments and converted them into percentage. Our results showed that G1 generation females laid eggs were 26% compared to control. In G2 and G3 generations, female laid eggs were 35.26% and 50.26%, respectively as compared to control (Fig. 1). While in G1-G3 control treatments, females were laid eggs 90%, 88%, and 90%, respectively. Therefore, these results indicated that the fecundity decreased over the generations with all being significantly lower than the control.

### Effect of pRNAi on hatched eggs

**Figure 2:**
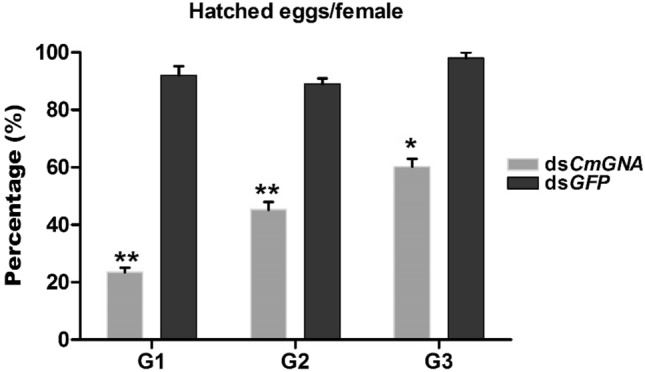
The percentage of hatched eggs per female were observed in G1–G3 generations. Each point indicates the mean ± standard error in G1–G3 generations and their control groups. Significant differences indicated by *(P < 0.05), **(P < 0.01).

After counting the laid eggs per female, oviposition box were placed in artificial chamber. Within in few days, 1st instar larvae were collected from hatched eggs. Newly emerged larvae were calculated and compared with previous counted eggs. Percentage hatched eggs per female were recorded as 23.53%, 45.26%, and 60.26% in G1–G3 generations, respectively as compared to control (Fig. 2). However, in control groups, hatched eggs were calculated 92%, 89%, and 98% in in G1-G3 generations, respectively. Therefore, our results suggested that hatched eggs were high significantly less in G1 and G2 generations and significantly less in G3 generation as compared to control treatments.

### Effect of pRNAi on larval mortalities

**Figure 3:**
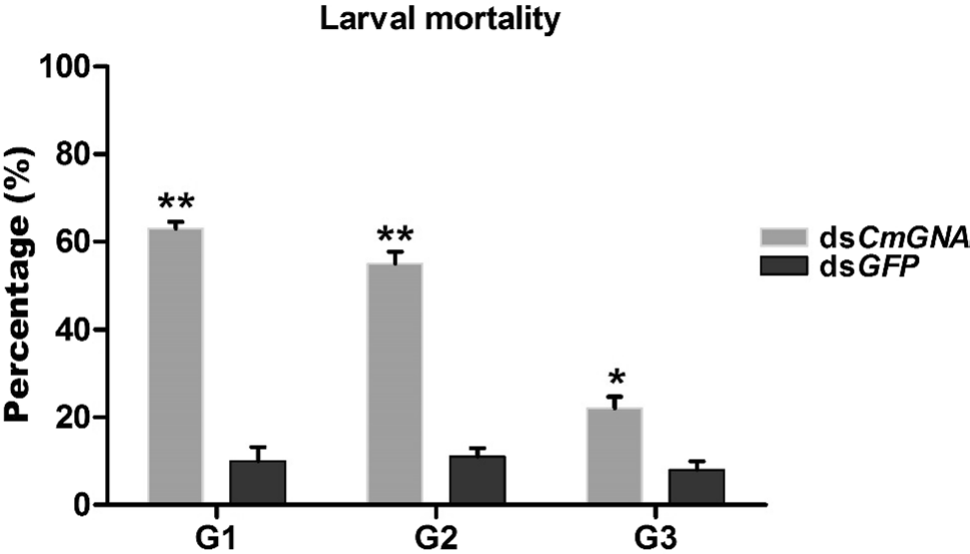
The percentage of larval mortalities were observed in G1–G3 generations. Each point indicates the mean ± standard error in G1–G3, and their control groups. Significant differences indicated by *(P < 0.05), **(P < 0.01).

Counted 1st instar larvae were collected and placed on fresh seedling of ShuHui-527 Chinese rice cultivar. Larval mortalities were recorded on regular bases until they become pupae. Larval mortifies were 63%, 55% and 22% in G1–G3 generations, respectively (Fig. 3). While in control treatments, larval deaths were noticed as 10%, 11% and 8% in G1–G3 generations, respectively. Herein, our results showed that larval mortalities were highly significant in G1 and G2 and significant in G3 generation.

### Effect of pRNAi on pupal mortalities

**Figure 4:**
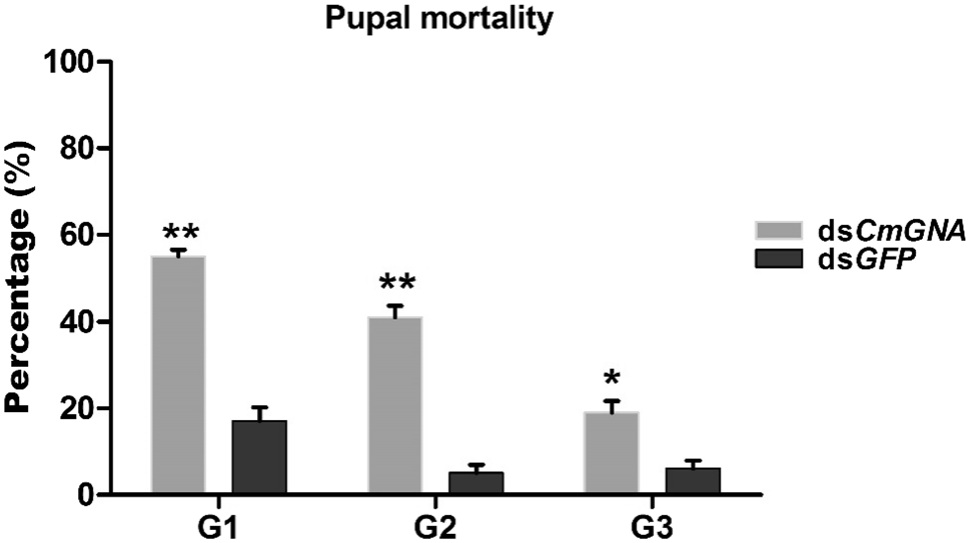
The percentage of dead pupae were calculated in G1–G3 generations. Each point indicates the mean ± standard error in G1–G3, and their control groups. Significant differences indicated by *(P < 0.05), **(P < 0.01).

Survived larvae in G1–G3 generations were counted, kept in rearing chamber, and allowed them to become pupae. Pupae growth were stunted, and with lack of formation of pupation chamber which led them into delayed life span. Several pupae were died in pupation chamber and did not emerge into adults. Pupal mortalities were 55, 41, and 19%, in G1, G2 and G3 generations, respectively (Fig. 4). In control treatment, pupal mortalities were 17%, 5%, and 6%, respectively. Our results suggested that pupal mortalities were highly significant in G1 and G2, while significant in G3 group compared to their controls.

### Effect of pRNAi on the rate of male emergence

**Figure 5:**
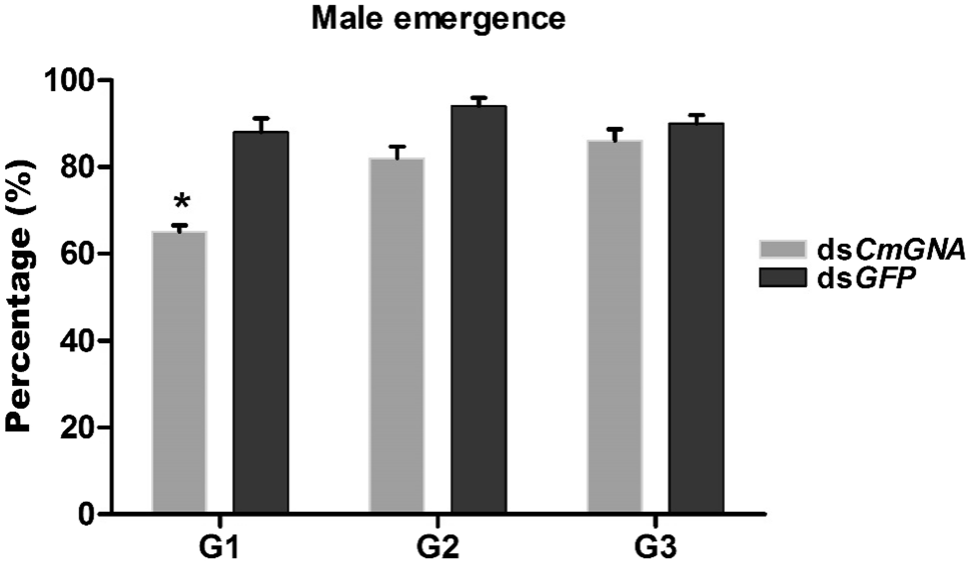
The percentage of emerged males from pupation chamber were calculated in G1–G3 genrations. Each point indicates the mean ± standard error from G1–G3 generations, and their control groups. Significant differences indicated by *(P < 0.05), **(P < 0.01).

In order to analyze the male emergence rate in G1–G3 ds*CmGNA* treated generations as compared to control, survived pupae were kept in rearing chamber until they become adults. Newly emerged adults were separated based on sexes. Male and female were counted in order to examine the rate of male emergence. In ds*CmGNA* treatments, emerged males in G1–G3 generations were recorded 65%, 82%, and 86%, respectively as compared to control (Fig. 5). However, male adults were emerged in ds*GFP* treatments were 88%, 94% and 90% in G1-G3 generations, respectively**.** These results indicated that male emergence was significantly different in only G1 generation as compared to control.

### Effect of pRNAi on the rate of female emergence

**Figure 6:**
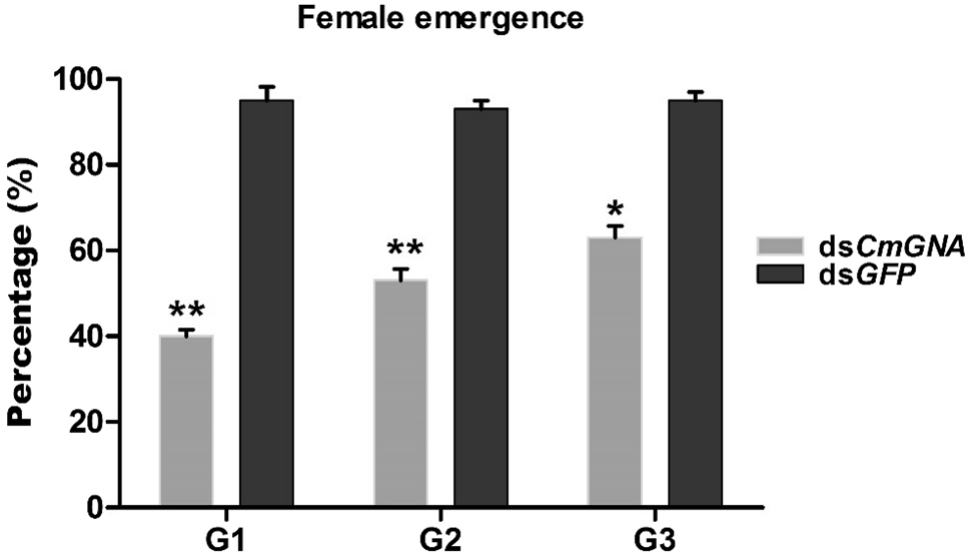
The percentage of emerged females after pupation. Each point indicates the mean ± standard error in G1–G3, and their control groups. Significant differences indicated by *(P < 0.05), **(P < 0.01).

In ds*CmGNA* treated G1–G3 generations, female adults were calculated as 40%, 53%, and 63% respectively as compared to control (Fig. 6), however, in control treatments, female adults were recorded as 95%, 93%, and 95%, respectively. These results indicated that female emergence was highly significant in G1 and G2, and significant in G3 generations as compared to control groups.

### Phenotypic deformities of pRNAi in G1–G3 generations

**Figure 7:**
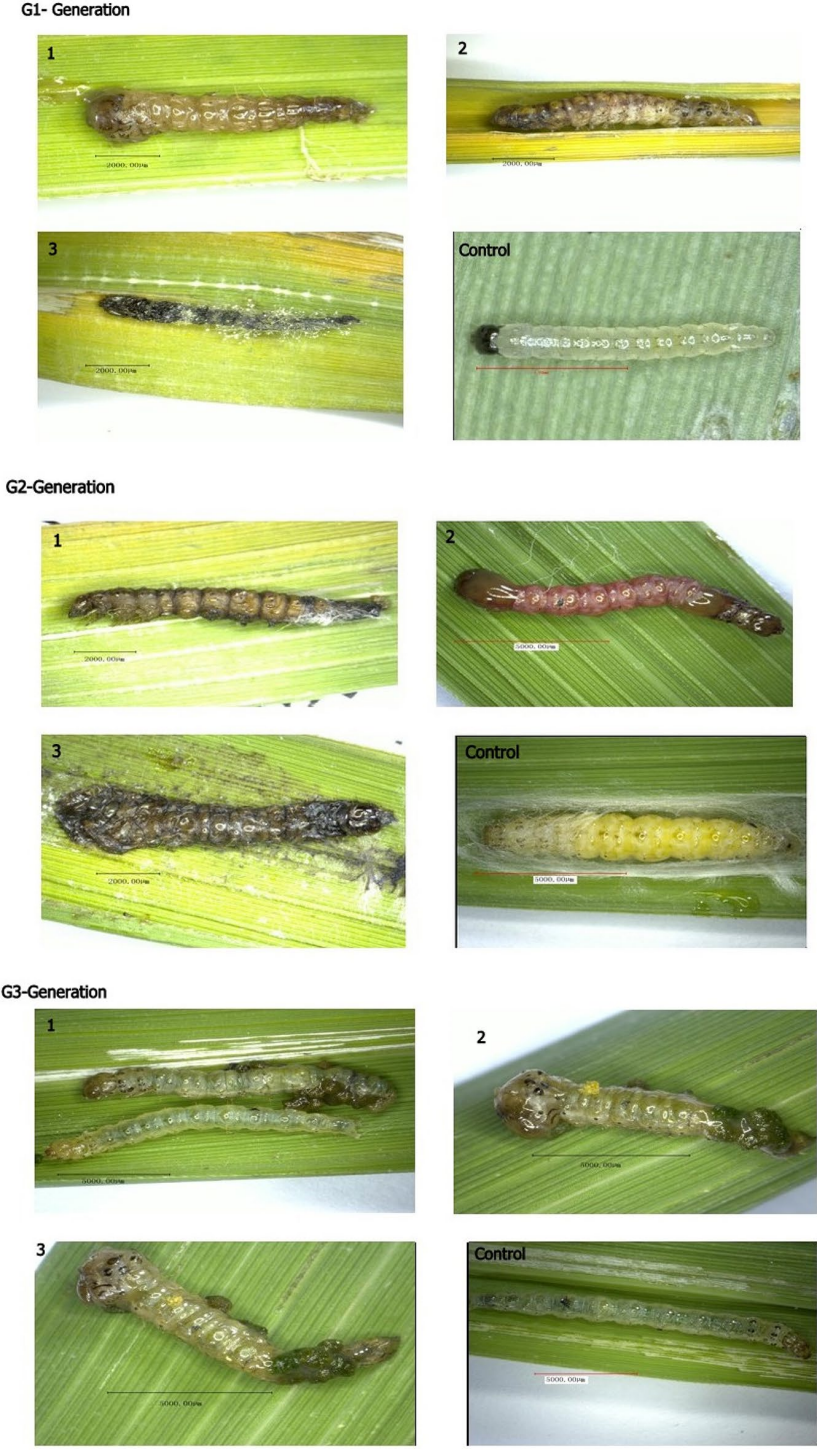
The phenotypic deformities were evaluated from larvae and pupae in G1–G3 generations**.** Infected larvae were observed in G1–G3 generations of treated insects using pRNAi.

**Figure 8:**
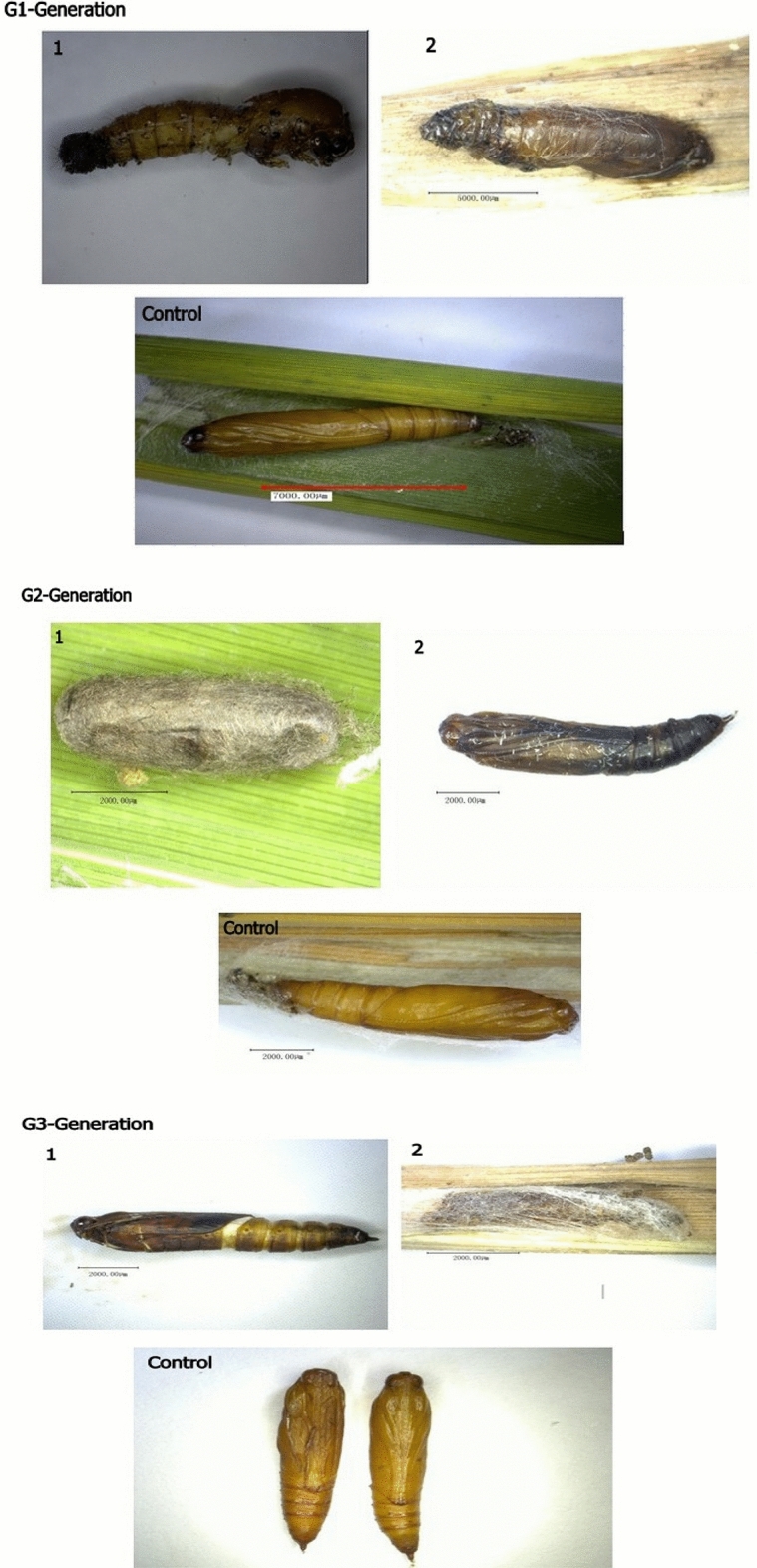
Pupae of treated insects exhibiting deformities in G1–G3 generations.

pRNAi effects of *CmGNA* on *C. medinalis* have been studied in G1–G3 generations. We observed that phenotypic deformities were present in both larvae and pupae. Our results indicated that larvae showed stunted growth, deformed shaped, and did not undergo in complete molting (Fig. 7). In contrast, no phenotypical deformities were examined in control treatments. Pupae did not emerged into adults and died in pupation chamber, while, significant percentage of pupae were emerged into adult individuals in control treatments (Fig. 8).

### Effect of pRNAi on *CmGNA* transcriptional level in G1–G3 generations

**Figure 9:**
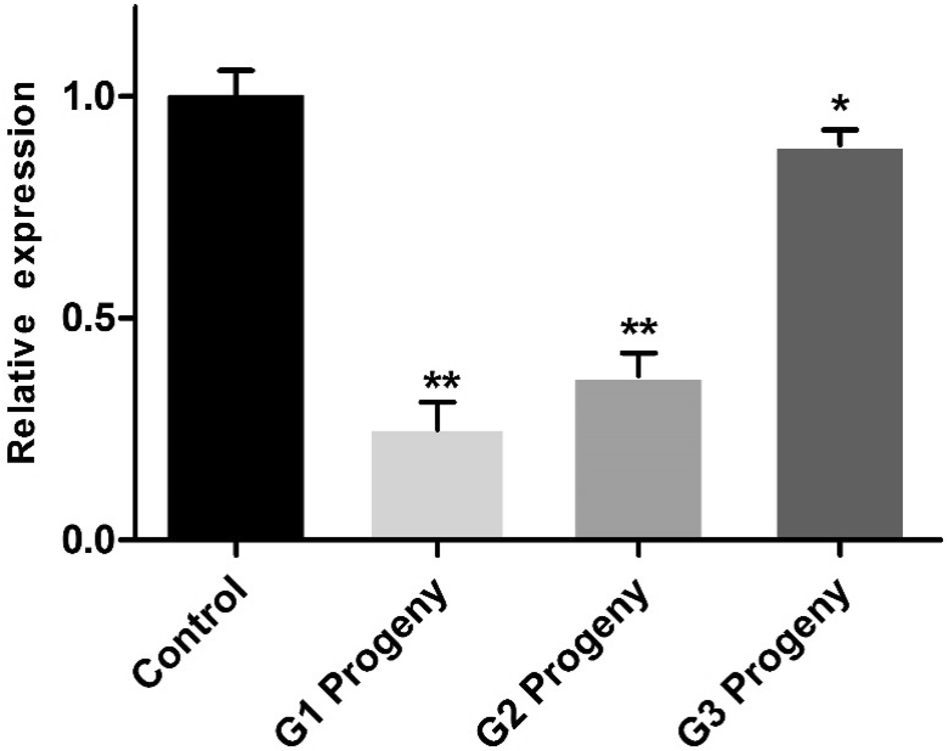
Changes in mRNA transcript level of *CmGNA* gene in G1–G3 generations after pRNAi. Each bar indicated the mean ± *SD*, and significant differences indicated by *(P < 0.05), **(P < 0.01).

Adult females were collected in G1–G3 generations, and used them to analyze the mRNA expression of *CmGNA*. We observed that mRNA expression level was high significanlty decreased in G1 and G2, while, signigicantly reduced in G3 generations (Fig. 9). However, there was no effcets on mRNA expression in G1–G3 generations of control treatments.

## Discussion

Insect pests consider an alarming threat to globe crop production, pant biodiversity, and animal and human health^93^. Synthetic chemicals are widely used for their control; however, insect pest resistance and resurgence, and off-target insect species has driven attention for searching alternative methods of insect pest control^8^. *Bt* (*Bacillus thuringiensis*) crops varieties containing insecticidal proteins bas been largely successful against lepidopteran insects^94^. Multiple cases reported that insect pest have become resistant against *Bt* varieties^95–97^. However, RNAi technology has been used against most different insect’s orders, such as Diptera, Hemiptera, Coleoptera, Lepidoptera, and Orthoptera^98–100^. Therefore, we used RNAi technology to target *C. medinalis*.

In this research work dsRNA corresponding to *CmGNA* gene were used to investigate the parental effect in *C. medinalis*. GNA is a key enzyme in insect biosynthesis pathway. We observed the pRNAi effects using dsRNA of *CmGNA* gene in *C. medinalis*. The G1-G3 generations were effected using ds*CmGNA* for in a long lasting manner. The experiments carried out with *C. medinalis* allowed us to measure the biological parameters and quantify the mRNA expression levels that continuously produces pRNAi in G1–G3 generations. Previous studies have demonstrated highly sensitive lethal pRNAi response in laid eggs of *C. medinalis*^67^. In the aforementioned studies, almost similar pRNAi method was used. We observed that reduction in laid eggs were significant in ds*CmGNA* treatments in three generations. Abiotic factors have great influence on life cycle history of insects. Among them, temperature is the most important factor that exerts effects on the biology, reproduction, and abundance of insects^101^. Therefore, we consider the decreased percentage biological parameters in control treatments may be due to different abiotic factors in our insect rearing chamber. Recently, it was studied that pRNAi could cause significant reduction of hatched eggs in G1-G3 generations of *C. medinalis*^67^. Reduction of hatched eggs observed in *D. virgifera virgifera* were due to lack of embryonic development in eggs^64^. pRNAi effects were also present in hatched eggs of *Nephotettix cincticeps*^94^. Our result also stated the significant reduction of hatched eggs which could be possible due to lack of embryonic development in eggs of *C. medinalis* G1–G3 generations^67^.

Insect’s larvae are a devastative for agricultural crops. At larval stage, larvae feed on plant’s parts that ultimately reduces the crop yield. *C. medinalis* single larva can damage multiple leaves and interfering with photosynthesis^102^. In a previous research, larval mortalities were observed in several insect pests using pRNAi^103^. We also described that pRNAi caused a significant larval mortality in G1-G3 generations of *C. medinalis*^67^. In the present research, our results also showed significant larval mortalities in G1–G3 generations of *C. medinalis*. Previously, researcher described that pRNAi showed pupal mortality resulted in knockdown of zygotic genes in offspring embryos^104^. Pupal mortalities were significantly observed in G1–G3 generations of *C. medinalis*^67^. Herein, pupal mortalities were significant in three generations of *C. medinalis*. Earlier studies have shown the highly sensitive and lethal pRNAi effects in *D. virgifera virgifera*^98^. However, stronger pRNAi effects were observed in female adults as compared to male^98^. Same results also were observed in adults of *C. medinalis*^67^. Our findings also suggested that pRNAi effects of *CmGNA* also caused significant effects in female as compared to male in G1–G3 generations. Therefore, *CmGNA* gene is suitable candidate for control of *C. medinalis* population.

DsRNA-degrading enzymes (dsRNases) have been considered as crucial factors reducing RNAi efficiency in many insect species. The presence of dsRNase have been studied in *B*. *mori* in which dsRNase is present is midgut and digestive juice^105^. Subsequently, dsRNases are present in many insects, such as *A*. *pisum*^106^, *M*. *sexta*^41^, *S*. *gregaria*^107^, *Spodoptera frugiperda*^108^, *P*. *xylostella*^109^, and *Lygus lineolaris*^110^. Previously, pRNAi effects were decreased gradually in G1–G3 generations of *C. medinalis*^67^. In this work, we also observed that pRNAi effects were highly significant in G1, significant in G2, and less significant in G3 generations. The mRNA expression was also decreased from G1–G3. According to our research outcomes, we considered that dsRNases may be present in *C. medinalis* that reduced the pRNAi efficiency. Due to presence of dsRNases, this research is not applicable at field level. Therefore, our future research direction will be analyze, characterize, and silence dsRNases efficiency that could be helpful in enhancing the effects of pRNAi and used against insecticide resistance in *C. medinalis*.

## Conclusion

In conclusion, we analyzed the pRNAi effects of *CmGNA* in the different developmental stages of *C. medinalis*. Herein, we have described that pRNAi of *CmGNA* reduced the population of this notorious insect pest at any developmental stage. Our investigation led the researcher to understand the crucial role of pRNAi in insect pest management strategies. These findings provide a framework of pRNAi for testing on plants. A way leads for pRNAi as insect pest management tool which help to observe the longevity of pRNAi effects. In addition, pRNAi of *CmGNA* also provide a platform to better understand pRNAi in different lepidopteron insects.

## Supplementary Information

Supplementary Information.
