# Supplementary material for: Glucosamine-6-phosphate N-acetyltransferase gene silencing by parental RNA interference in rice leaf folder, Cnaphalocrocis medinalis (Lepidoptera: Pyralidae)
Source: Sci Rep. 2022 Feb 8;12:2141. doi: 10.1038/s41598-022-06193-9 (PMC8825807; doi:10.1038/s41598-022-06193-9)
Supplement: Supplementary file 1 — Supplementary Information. [file 41598_2022_6193_MOESM1_ESM.docx]

**Glucosamine-6-phosphate N-acetyltransferase Gene Silencing by Parental RNA Interference in Rice Leaf Folder, Cnaphalocrocis medinalis (Lepidoptera: Pyralidae)**

Muhammad Shakeel ^1^, Juan Du^1^, Shang-Wei Li^1*^, Yuan-Jin Zhou^1^, Naeem Sarwar^2^, and Xiaolan Guo^3^

^1^ Provincial Key Laboratory for Agricultural Pest Management of Mountainous Regions, Institute of Entomology, Guizhou University, Guiyang, Guizhou, 550025, China.

^2^ Department of Agronomy, Bahauddin Zakariya University, Multan, 60800, Pakistan.

^3^ College of Forestry, Guizhou University, Huaxi, 550025,

* Correspondence to [shangwei65@aliyan.com](mailto:shangwei65@aliyan.com)**.**


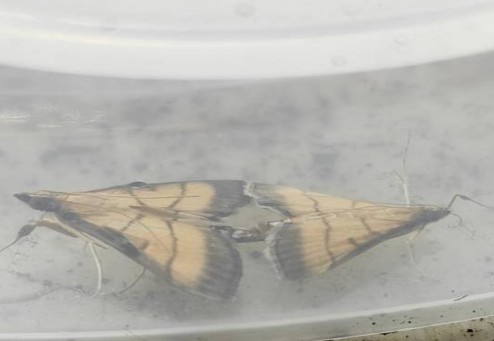


**Fig. S1. Mating of paired adult of *C. medinalis* in a oviposition box**

**
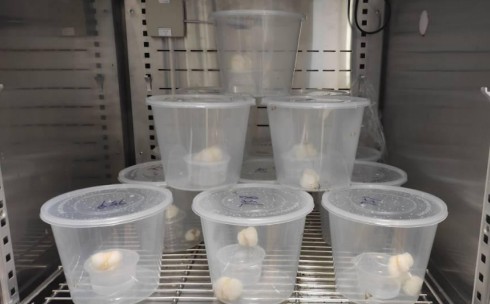
**

**Fig. S2. Feeding and oviposition of paired adults of *C. medinalis* in a oviposition box. Laid eggs were observed inside the wall of lid and box.**


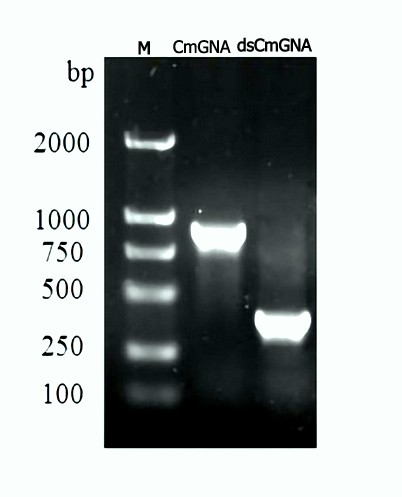


**Figure S3:** RT-PCR results indicated cDNA sequence length of *CmGNA and* ds*CmGNA* length of *C. medinalis*.

**
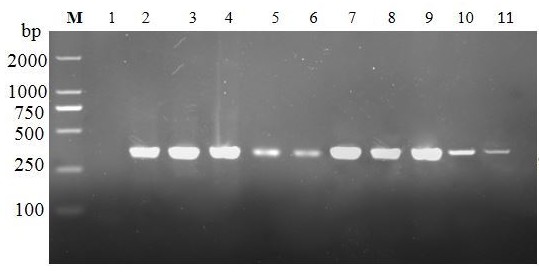
**

**Fig. S4. Electrophoresis of dsRNA synthesis**

**2-6:** ds*CmGNA.* **7-11:** ds*GFP*. **M:** DL2000 DNA marker.

**Table S1.** Primer information for cloning and expression analysis of *CmGNA*

| **Primer Name** | **Primer Sequence** | **Primer Usage** |
| --- | --- | --- |
| GNA-F | TTCGAAGTTGGGCGATAGGTG | RT-PCR |
| GNA-R | TGTTGTGGTGATGCGAAGTCT |  |
| GNA-iF | AGTACCTCTACCCCCCAGATATCC | ds*CmGNA* sequence amplification  ds*CmGNA* Synthesis    ds*GFP* sequence amplification  ds*GFP* Synthesis |
| GNA -iR | TATAAGTGTCATTGACTACGACGT |  |
| GNA -dsF | taatacgactcactatagggAGTACCTCTACCCCCCAGATATCC |  |
| GNA -dsR | taatacgactcactatagggTATAAGTGTCATTGACTACGACGT |  |
| GFP -iF | GCCAACACTTGTCACTACTT |  |
| GFP -iR | GGAGTATTTTGTTGATAATGGTCTG |  |
| GFP -dsF | taatacgactcactatagggGCCAACACTTGTCACTACTT |  |
| GFP -dsR | taatacgactcactatagggGGAGTATTTTGTTGATAATGGTCTG |  |
| GNA -qF | GTGAAGACTGGATGGTGGTG | RT-qPCR |
| GNA -qR | TCGGCGCGGCTACCCTCACG |  |
| Actin-F | ATGGTCGGCATGGGACAG |  |
| Actin-R | GAGTTCATTGTAGAAGGTGT |  |

**Note:** The lowercase letters in the primers indicate the T7 promoter sequence.
